# Synthesis and structure of 4-[(2,3,4,5,6-pentafluoro­phen­oxy)carbon­yl]phenyl 4-(dec­yloxy)benzoate

**DOI:** 10.1107/S2056989026007759

**Published:** 2026-08-04

**Authors:** Khaleel Ahmed, G. N. Venkata Reddy, B. Bommalingaiah, B. S. Palakshamurthy, H. T. Srinivasa, H. C. Devarajegowda

**Affiliations:** a Department of Physics Yuvaraja's College University of Mysore,Mysore-570005 Karnataka India; b Department of Physics, Government Science College, Chithradurga-577501, Kanataka, India; c Department of PG Studies and Research in Physics Albert Einstein Block UCS Tumkur University, Tumkur Karnataka-572103 India; d Raman Research Institute, C V Raman Avenue Sadashivanagar Bangalore Karnataka India; e Department of Physics Yuvaraja's College University of Mysore,Mysore-570005 Karnaataka India; University of Aberdeen, United Kingdom

**Keywords:** crystal structure, (dec­yloxy)benzoate, Hirshfeld surface, 2,3,4,5,6-penta-fluoro­phen­oxy group

## Abstract

In the title compound, C_30_H_29_F_5_O_5_, the dihedral angle between the central carbonyl­phenyl and peripheral perfluoro­phen­oxy and benzoate rings are 76.57 (2) and 69.71 (3)°, respectively. The pendant C10 alkyl chain adopts an all-*anti* conformation. In the crystal, weak C—H⋯O hydrogen bonds connect the mol­ecules, generating [010] *C*(7) chains. The packing is consolidated by C—H⋯F and C—H⋯π inter­actions and weak aromatic π–π stacking. A Hirshfeld surface analysis revealed that the major contributions to the packing are from H⋯H (42.2%), H⋯F/F⋯H (19.0%), H⋯O/O⋯H (10.3%), C⋯H/H⋯C (8.7%) and F⋯C/C⋯F (8.0%) contacts.

## Abstract 2

In the title compound, the dihedral angles between the central aromatic ring and its peripheral rings are are 76.57 (2) and 69.71 (3)° and the C10 alkyl chain adopts an extended conformation.

## 1. Chemical context

The family of aromatic ester derivatives incorporating a *para*-dec­yloxy substituent attached to a benzoate core constitute an important class of organic compounds due to their wide-ranging applications in liquid-crystalline materials, supra­molecular assemblies, optoelectronic devices and functional organic systems. The incorporation of long flexible alk­oxy chains into rigid aromatic ester frameworks significantly influences mol­ecular anisotropy, inter­molecular inter­actions and mesophase organization, thereby promoting ordered mol­ecular packing and thermal stability (Muchenedi & Madhu Mohan, 2020 ▸). In particular, dec­yloxy-substituted benzoate derivatives have attracted considerable attention because the extended alkyl chain enhances van der Waals inter­actions and contributes to improved mol­ecular ordering, mesophase stability and thermo-responsive behaviour (Ashmawy *et al.*, 2022 ▸). Phenyl 4-(dec­yloxy)benzoate belongs to the family of calamitic liquid-crystalline aromatic esters containing a rigid phenyl benzoate core connected to a flexible dec­yloxy substituent in the para position. Such mol­ecular architectures are well known for exhibiting structural anisotropy together with favourable inter­molecular π–π stacking inter­actions between aromatic rings and hydro­phobic inter­actions involving the long alkyl chain. These features strongly influence crystal packing arrangements, thermal transitions and mesomorphic properties (Das *et al.*, 2012 ▸).

As part of our ongoing studies on these systems (Ahmed *et al.*, 2026*a* ▸,*b* ▸), we present the synthesis and crystal structure of the title compound, C_30_H_29_F_5_O_5_ (**I**), herein.
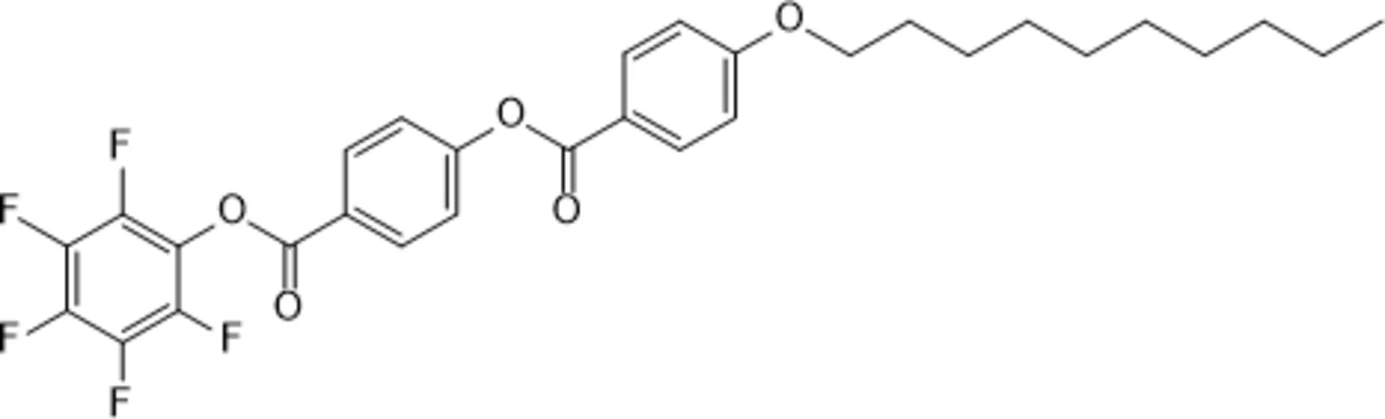


## 2. Structural commentary

**Figure 1:**
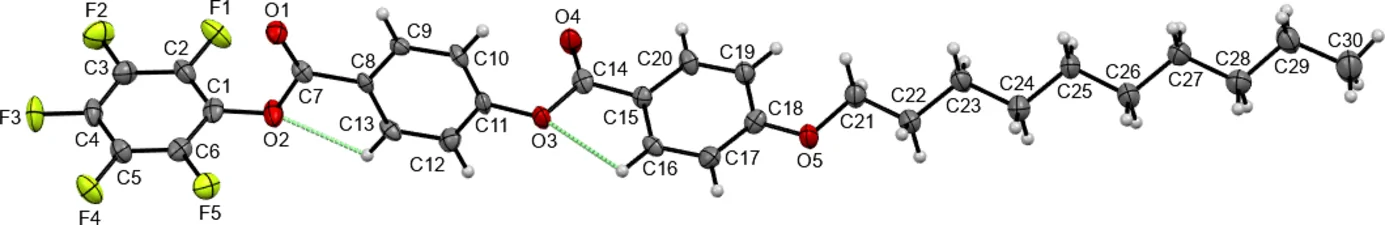
The mol­ecular structure of (**I**) showing 50% probability ellipsoids and short C—H⋯O intra­molecular contacts as green dotted lines.

The mol­ecular structure of (**I**) is shown in Fig. 1 ▸. The dihedral angles between the central carbonyl­phenyl (C8–C13) ring and pendant perfluoro­phen­oxy (C1–C6) and (dec­yloxy)benzoate (C15–C20) rings are 76.57 (2) and 69.71 (2)°, respectively, thus the central ring is close to normal to both peripheral rings. The C7/O1/O2 carboxyl­ate group is twisted from its attached C8–C13 ring by 3.5 (5)° and the corresponding value for the C14/O3/O4 group and the C15–C20 ring is 4.5 (6)°. The pendant C10 alkyl chain adopts an all-*anti* conformation with the smallest and largest torsion angles being −107.17 (3)° and −178.34 (3)°, respectively, close to the ideal ±180°. Two short intra­molecular C—H⋯O contacts (Table 1 ▸) are observed. Otherwise the bond lengths and angles in (**I**) may be regarded as normal.

## 3. Supra­molecular features

**Figure 2:**
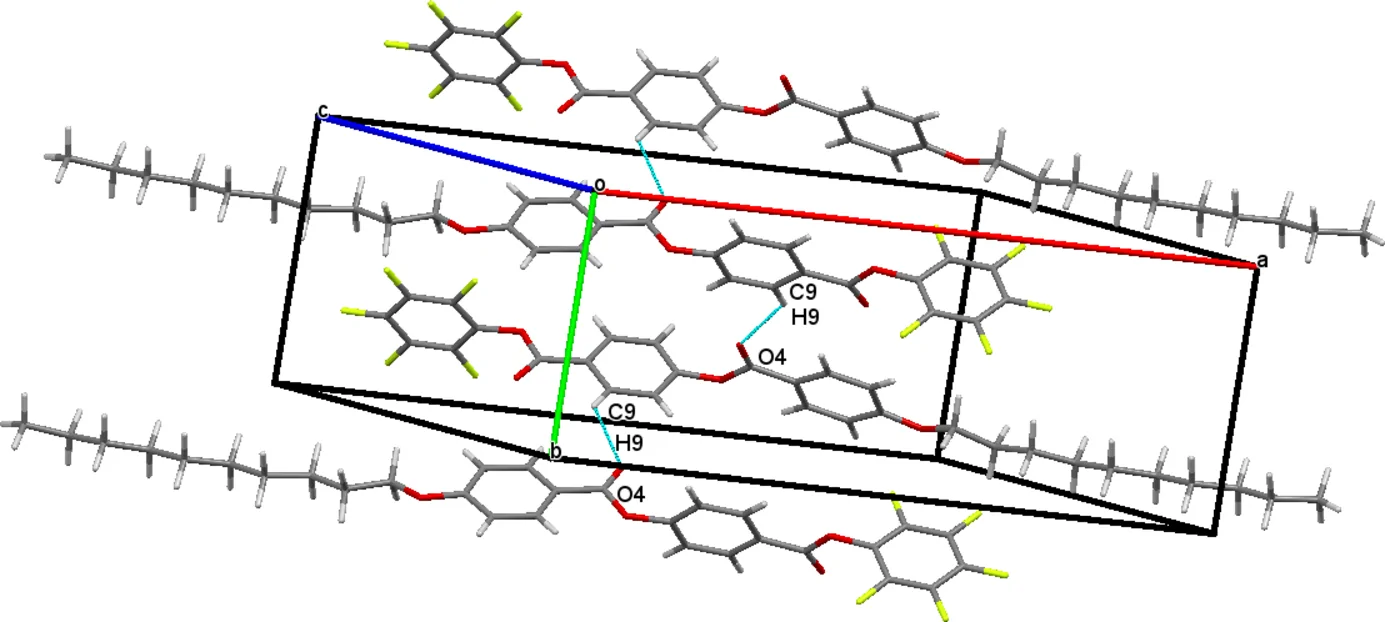
Detail of the extended structure of (**I**) showing C—H⋯O hydrogen bonds (grey dotted lines) connecting the mol­ecules into *C*(7) chains.

**Figure 3:**
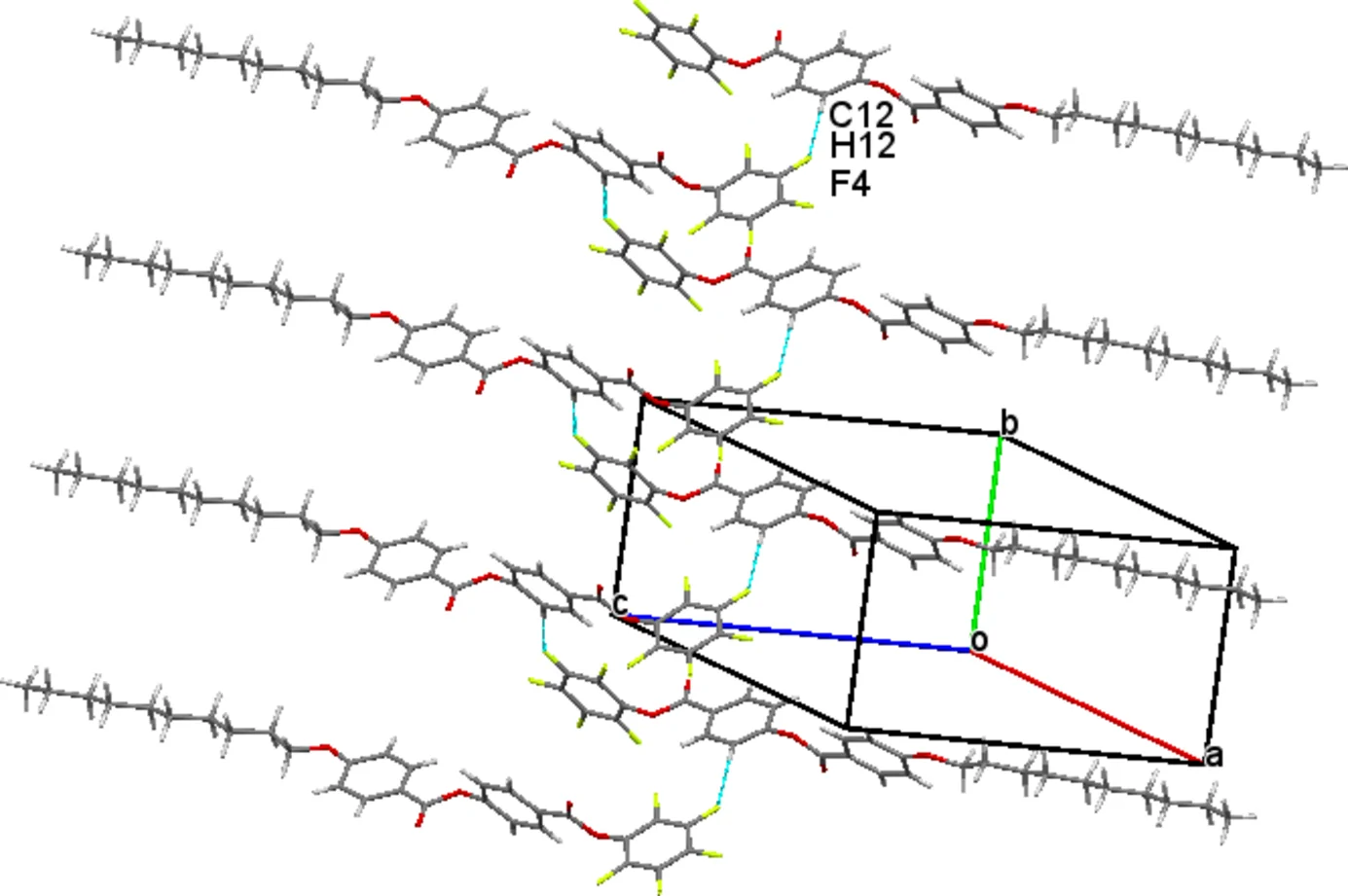
Detail of the extended structure of (**I**) showing C—H⋯F hydrogen bonds (grey dotted lines) connecting the mol­ecules into *C*(10) chains.

**Figure 4:**
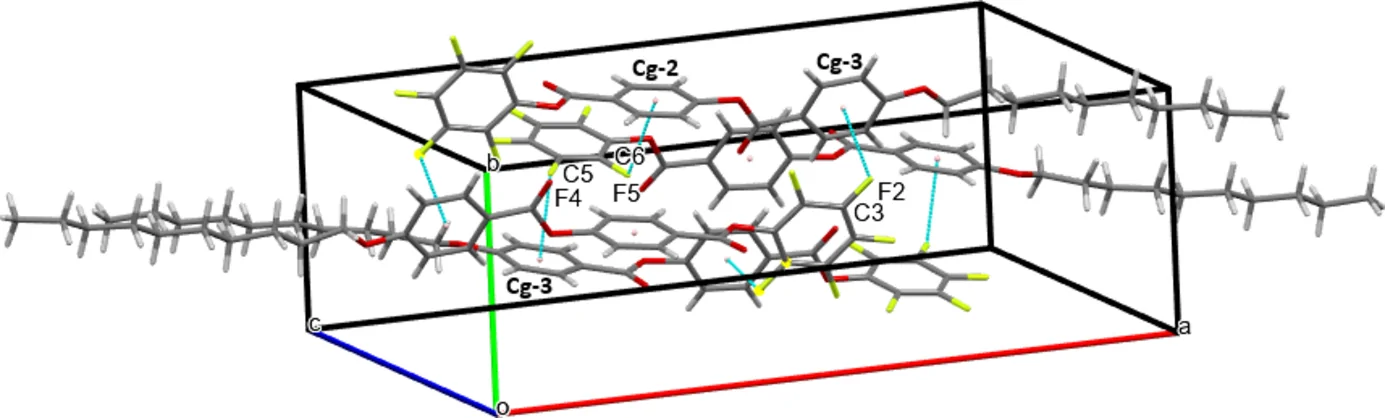
Detail of the extended structure of (**I**) showing C—F⋯π inter­actions.

**Figure 5:**
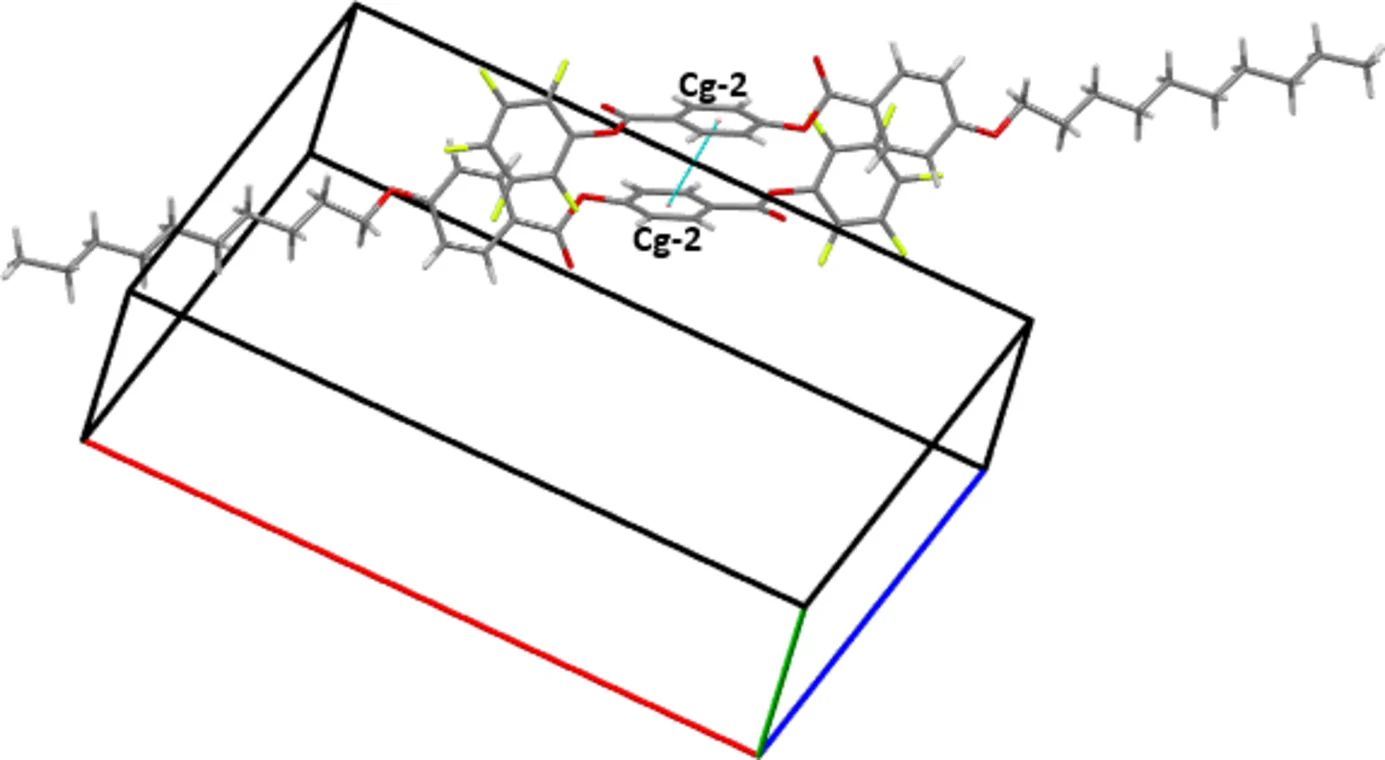
Detail of the extended structure of (**I**) showing π–π stacking inter­actions.

In the extended structure, the mol­ecules of (**I**) are connected by weak C9—H9⋯O4 hydrogen bonds to generate a *C*(7) chain propagating in zigzag fashion along [010] as shown in Fig. 2 ▸. The chain is reinforced by C12—H12⋯F4 hydrogen bonds, which lead to a *C*(10) chain motif (Fig. 3 ▸). The packing is consolidated by C—F⋯π inter­actions namely C3—F2⋯*Cg*3, C5—F4⋯*Cg*3 and C6—F5⋯*Cg*2, where *Cg*3 and *Cg*2 are the centroids of the C15–C20 and C8–C13 rings, respectively (Fig. 4 ▸). A weak aromatic π–π stacking inter­action between pairs of *Cg*2 rings related by inversion symmetry (1 − *x*, 1 − *y*, −*z*) with a centroid–centroid distance of 3.918 (4) Å and a slippage of 1.830 Å is observed and is shown in Fig. 5 ▸.

## 4. Hirshfeld surface analysis

**Figure 6:**
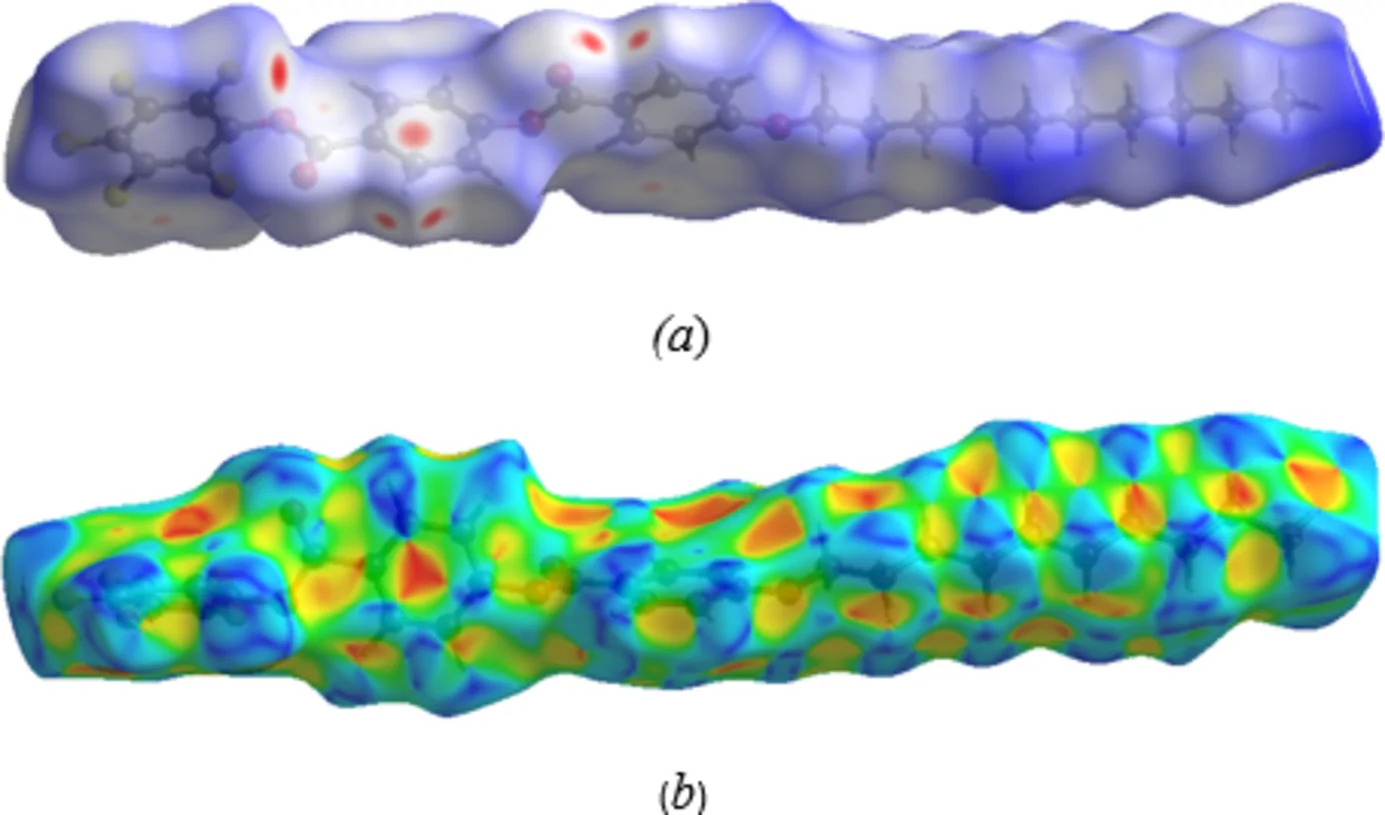
View of the three-dimensional Hirshfeld surface of (**I**) plotted over (*a*) *d*_norm._ and (*b*) shape-index.

**Figure 7:**
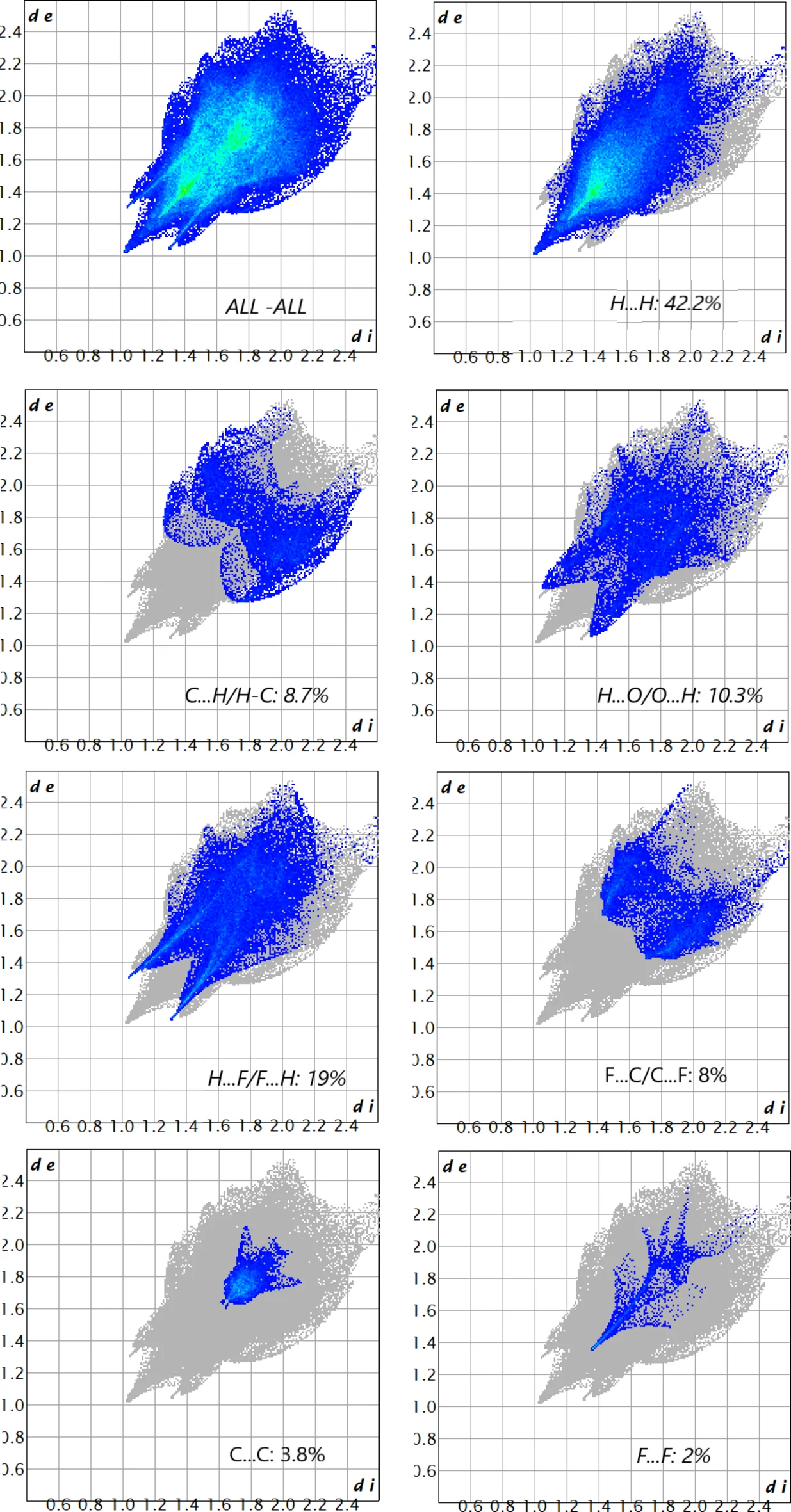
The two- dimensional fingerprint plots of (**I**) showing the contributions from the different contact types.

A Hirshfeld surface (HS) analysis for (**I**) was performed using *CrystalExplorer* (Spackman *et al.*, 2021 ▸) and the HS mapped over *d*_norm_ and shape-index are illustrated in Fig. 6 ▸. The red triangular region viewed normal to the centre of carbophenyl ring indicates the existence of π–π stacking. The two-dimensional fingerprint plots shown in Fig. 7 ▸ indicate that the major contributions to the HS for (**I**) arise from H⋯H (42.2%), H⋯F/F⋯H (19.0%), H⋯O/O⋯H (10.3%), C⋯H/H⋯C (8.7%), F⋯C/C⋯F (8.0%) contacts, with C⋯C (3.8%) and F⋯F (2.0%) contacts playing a smaller role.

## 5. Database survey

A search of the Cambridge Structural Database (CSD version 6.01, March 2026; Groom *et al.*, 2016 ▸) for structures containing the 4-(dec­yloxy)benzoate moiety yielded five related entries, namely CSD refcodes LASLIE (Kazem-Rostam *et al.*, 2019 ▸), PUWQOP (Dutronc *et al.*, 2016 ▸), ROJWOE and ROJWUK (Kuz’Mina *et al.*, 2014 ▸) and TUVBUI (Cheng *et al.*, 2010 ▸). The dihedral angles between the substituent planes and the 4-(dec­yloxy) benzoate moiety in these structures are 66.7, 58.0, 58.4, 64.6 and 48.5°, respectively, compared to a value of 69.71 (3) ° for (**I**).

## 6. Synthesis and crystallization

A mixture of 2,3,4,5,6-penta­fluoro­phenol (0.184 g, 1 eq.) and 4-((4- (dec­yloxy)benzo­yl)­oxy)benzoic acid (0.398 g, 1 eq.) in di­chloro­methane was stirred at room temperature overnight using di­cyclo­hexyl­carbodi­imide (1.2 eq) to promote the esterification reaction in the presence of *N*,*N*-di­methyl­amino­pyrimidine as a catalyst. The insoluble byproduct, di­cyclo­hexyl urea, was removed by filtration. The filtrate was washed with 5% acetic acid solution in water, and then with pure water. The filtrate was passed through silica gel, and then left for a week to grow crystals for X-ray studies. ^1^H NMR (500 MHz, CDCl 3) : δ 8.12–8.02 (*m*, 4H, Ar-H), 7.54 (*m*, 2H, Ar-H), 7.10 (*d*, *J* = 8.5 Hz, 2H, Ar-H), 4.01 (*t*, *J* = 6.5 Hz, 2H, –OCH_2_–), 1.74–1.25 (*m*, 16H, –CH_2_–alk­yl), 0.91 (*t*, *J* = 4.5Hz, 3H, –CH_3_) ppm. Elemental analysis calculated: C, 63.83; H, 5.18; F, 16.83%; found C, 63.90; H, 5.15; F, 16.79%.

## 7. Refinement

Crystal data, data collection and structure refinement details are summarized in Table 2 ▸. All H atoms were positioned with idealized geometry and refined using a riding model with C—H = 0.95–0.99 Å and *U*_iso_(H) = 1.2*U*_eq_(C) or 1.5*U*_eq_(methyl C).

## Supplementary Material

Crystal structure: contains datablock(s) I. DOI: 10.1107/S2056989026007759/hb8232sup1.cif

Structure factors: contains datablock(s) I. DOI: 10.1107/S2056989026007759/hb8232Isup3.hkl

Supporting information file. DOI: 10.1107/S2056989026007759/hb8232Isup3.cml

CCDC reference: 2576874

Additional supporting information:  crystallographic information; 3D view; checkCIF report

## Figures and Tables

**Table 1:** Hydrogen-bond geometry (Å, °) *Cg*3 and *Cg*2 are the centroids of the C15–C20 and C8–C13 rings, respectively.

*D*—H⋯*A*	*D*—H	H⋯*A*	*D*⋯*A*	*D*—H⋯*A*
C13—H13⋯O2	0.95	2.38	2.705 (6)	99
C16—H16⋯O3	0.95	2.42	2.737 (6)	99
C12—H12⋯F4^i^	0.95	2.48	3.427 (6)	174
C9—H9⋯O4^ii^	0.95	2.51	3.175 (7)	127
C3—F2⋯*Cg*3^iii^	1.340 (6)	3.242 (4)	3.630 (6)	95.8 (3)
C5—F4⋯*Cg*3^iv^	1.337 (6)	3.106 (4)	3.403 (6)	91.0 (3)
C6—F5⋯*Cg*2^v^	1.336 (6)	3.446 (4)	4.011 (6)	105.3 (3)

Symmetry codes: (i) 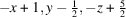; (ii) 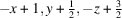; (iii) 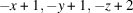; (iv) 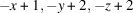; (v) 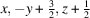.

**Table 2:** Experimental details

Crystal data	
Chemical formula	C_30_H_29_F_5_O_5_
*M*_r_	564.53
Crystal system, space group	Monoclinic, *P*2_1_/*c*
Temperature (K)	143
*a*, *b*, *c* (Å)	23.579 (9), 8.255 (3), 13.959 (5)
β (°)	97.524 (10)
*V* (Å^3^)	2693.9 (17)
*Z*	4
Radiation type	Mo *K*α
μ (mm^−1^)	0.12
Crystal size (mm)	0.33 × 0.28 × 0.18

Data collection	
Diffractometer	Bruker SMART APEXII CCD
Absorption correction	Multi-scan (*SADABS*; Krause et al., 2015 ▸)
*T*_min_, *T*_max_	0.954, 0.970
No. of measured, independent and observed [*I* > 2σ(*I*)] reflections	12617, 4156, 2799
*R*_int_	0.079
θ_max_ (°)	24.0
(sin θ/λ)_max_ (Å^−1^)	0.572

Refinement	
*R*[*F*^2^ > 2σ(*F*^2^)], *wR*(*F*^2^), *S*	0.091, 0.187, 1.09
No. of reflections	4156
No. of parameters	362
H-atom treatment	H-atom parameters constrained
Δρ_max_, Δρ_min_ (e Å^−3^)	0.29, −0.27

Computer programs: *APEX2* and *SAINT* (Bruker, 2017 ▸), *SHELXT2019/2* (Sheldrick, 2015*a* ▸), *SHELXL2019/2* (Sheldrick, 2015*b* ▸), *Mercury* (Macrae *et al.*, 2020 ▸) and *publCIF* (Westrip, 2010 ▸).

## supplementary crystallographic information

### 4-[(2,3,4,5,6-Pentafluorophenoxy)carbonyl]phenyl 4-(decyloxy)benzoate Crystal data

**Table:**

C_30_H_29_F_5_O_5_	Prism
*M**_r_* = 564.53	*D*_x_ = 1.392 Mg m^−^^3^
Monoclinic, *P*2_1_/*c*	Mo *K*α radiation, λ = 0.71073 Å
*a* = 23.579 (9) Å	Cell parameters from 4231 reflections
*b* = 8.255 (3) Å	θ = 2–26°
*c* = 13.959 (5) Å	µ = 0.12 mm^−^^1^
β = 97.524 (10)°	*T* = 143 K
*V* = 2693.9 (17) Å^3^	Prism, colourless
*Z* = 4	0.33 × 0.28 × 0.18 mm
*F*(000) = 1176	

### 4-[(2,3,4,5,6-Pentafluorophenoxy)carbonyl]phenyl 4-(decyloxy)benzoate Data collection

**Table:**

Bruker SMART APEXII CCD diffractometer	4156 independent reflections
Radiation source: fine-focus sealed tube	2799 reflections with *I* > 2σ(*I*)
Graphite monochromator	*R*_int_ = 0.079
Detector resolution: 1.09 pixels mm^-1^	θ_max_ = 24.0°, θ_min_ = 2.9°
ω scans	*h* = −26→26
Absorption correction: multi-scan (SADABS; Krause et al., 2015)	*k* = −9→8
*T*_min_ = 0.954, *T*_max_ = 0.970	*l* = −15→15
12617 measured reflections	

### 4-[(2,3,4,5,6-Pentafluorophenoxy)carbonyl]phenyl 4-(decyloxy)benzoate Refinement

**Table:**

Refinement on *F*^2^	Primary atom site location: structure-invariant direct methods
Least-squares matrix: full	Secondary atom site location: difference Fourier map
*R*[*F*^2^ > 2σ(*F*^2^)] = 0.091	Hydrogen site location: inferred from neighbouring sites
*wR*(*F*^2^) = 0.187	H-atom parameters constrained
*S* = 1.09	*w* = 1/[σ^2^(*F*_o_^2^) + (0.0315*P*)^2^ + 8.3116*P*] where *P* = (*F*_o_^2^ + 2*F*_c_^2^)/3
4156 reflections	(Δ/σ)_max_ < 0.001
362 parameters	Δρ_max_ = 0.29 e Å^−^^3^
0 restraints	Δρ_min_ = −0.27 e Å^−^^3^
0 constraints	

### 4-[(2,3,4,5,6-Pentafluorophenoxy)carbonyl]phenyl 4-(decyloxy)benzoate Special details

**Table:**

Geometry. All esds (except the esd in the dihedral angle between two l.s. planes) are estimated using the full covariance matrix. The cell esds are taken into account individually in the estimation of esds in distances, angles and torsion angles; correlations between esds in cell parameters are only used when they are defined by crystal symmetry. An approximate (isotropic) treatment of cell esds is used for estimating esds involving l.s. planes.

### 4-[(2,3,4,5,6-Pentafluorophenoxy)carbonyl]phenyl 4-(decyloxy)benzoate Fractional atomic coordinates and isotropic or equivalent isotropic displacement parameters (Å^2^)

**Table:**

	*x*	*y*	*z*	*U*_iso_*/*U*_eq_	
F1	0.31015 (14)	0.5559 (4)	1.0898 (2)	0.0496 (9)	
F2	0.22703 (13)	0.5716 (4)	1.2053 (2)	0.0507 (9)	
F3	0.24087 (13)	0.7618 (4)	1.3653 (2)	0.0496 (9)	
F4	0.33676 (13)	0.9403 (4)	1.4064 (2)	0.0423 (8)	
F5	0.41825 (12)	0.9326 (4)	1.2885 (2)	0.0397 (8)	
O1	0.36415 (14)	0.8802 (4)	1.0164 (2)	0.0308 (9)	
O2	0.41087 (14)	0.7276 (5)	1.1340 (2)	0.0372 (10)	
O3	0.60858 (13)	0.7810 (4)	0.8770 (2)	0.0300 (9)	
O4	0.57335 (14)	0.6245 (5)	0.7522 (2)	0.0366 (10)	
O5	0.81567 (13)	0.8385 (5)	0.6407 (2)	0.0356 (9)	
C1	0.3659 (2)	0.7414 (7)	1.1878 (4)	0.0285 (12)	
C2	0.3166 (2)	0.6550 (7)	1.1675 (4)	0.0335 (13)	
C3	0.2746 (2)	0.6603 (7)	1.2264 (4)	0.0335 (13)	
C4	0.2817 (2)	0.7555 (7)	1.3077 (4)	0.0322 (13)	
C5	0.3306 (2)	0.8456 (6)	1.3280 (4)	0.0291 (12)	
C6	0.3716 (2)	0.8405 (6)	1.2688 (4)	0.0279 (12)	
C7	0.4065 (2)	0.8078 (6)	1.0468 (3)	0.0236 (11)	
C8	0.45875 (19)	0.7889 (6)	1.0016 (3)	0.0215 (11)	
C9	0.46190 (19)	0.8706 (6)	0.9151 (3)	0.0222 (11)	
H9	0.429983	0.931497	0.886195	0.027*	
C10	0.51070 (19)	0.8639 (6)	0.8713 (3)	0.0252 (12)	
H10	0.512693	0.918672	0.811968	0.030*	
C11	0.55630 (19)	0.7772 (6)	0.9145 (3)	0.0258 (12)	
C12	0.5545 (2)	0.6922 (6)	0.9994 (3)	0.0291 (12)	
H12	0.586470	0.629745	1.026658	0.035*	
C13	0.5053 (2)	0.6994 (6)	1.0442 (3)	0.0285 (12)	
H13	0.503481	0.643826	1.103268	0.034*	
C14	0.6116 (2)	0.7045 (6)	0.7913 (4)	0.0276 (12)	
C15	0.66591 (19)	0.7375 (6)	0.7540 (3)	0.0242 (11)	
C16	0.70963 (19)	0.8289 (6)	0.8044 (3)	0.0246 (12)	
H16	0.705286	0.870860	0.866387	0.029*	
C17	0.7589 (2)	0.8585 (7)	0.7652 (4)	0.0323 (13)	
H17	0.788631	0.919849	0.800580	0.039*	
C18	0.76585 (19)	0.7992 (6)	0.6733 (4)	0.0258 (12)	
C19	0.7228 (2)	0.7059 (7)	0.6232 (4)	0.0302 (13)	
H19	0.727038	0.663152	0.561338	0.036*	
C20	0.6741 (2)	0.6764 (6)	0.6640 (4)	0.0294 (12)	
H20	0.644851	0.611809	0.629631	0.035*	
C21	0.8205 (2)	0.8072 (7)	0.5404 (4)	0.0335 (13)	
H21A	0.791261	0.870042	0.498526	0.040*	
H21B	0.814203	0.690676	0.526039	0.040*	
C22	0.8789 (2)	0.8558 (7)	0.5216 (4)	0.0344 (13)	
H22A	0.907479	0.788148	0.561637	0.041*	
H22B	0.885667	0.969954	0.541501	0.041*	
C23	0.8877 (2)	0.8384 (8)	0.4163 (4)	0.0365 (14)	
H23A	0.874505	0.729495	0.393464	0.044*	
H23B	0.863460	0.919194	0.377734	0.044*	
C24	0.9486 (2)	0.8604 (7)	0.3977 (4)	0.0363 (14)	
H24A	0.972565	0.776785	0.434192	0.044*	
H24B	0.962274	0.967530	0.422896	0.044*	
C25	0.9571 (2)	0.8495 (7)	0.2918 (4)	0.0347 (13)	
H25A	0.939578	0.747682	0.264592	0.042*	
H25B	0.936630	0.940687	0.256462	0.042*	
C26	1.0188 (2)	0.8536 (8)	0.2745 (4)	0.0384 (14)	
H26A	1.038810	0.759379	0.307209	0.046*	
H26B	1.036802	0.952824	0.304576	0.046*	
C27	1.0274 (2)	0.8509 (8)	0.1688 (4)	0.0375 (14)	
H27A	1.007572	0.754697	0.138124	0.045*	
H27B	1.008932	0.948067	0.136991	0.045*	
C28	1.0890 (2)	0.8467 (8)	0.1499 (4)	0.0408 (15)	
H28A	1.109513	0.940061	0.182805	0.049*	
H28B	1.107167	0.746625	0.178486	0.049*	
C29	1.0960 (2)	0.8521 (8)	0.0440 (4)	0.0435 (15)	
H29A	1.080660	0.956372	0.016903	0.052*	
H29B	1.072676	0.764400	0.010334	0.052*	
C30	1.1572 (2)	0.8342 (9)	0.0223 (4)	0.0516 (17)	
H30A	1.158467	0.851192	−0.046847	0.077*	
H30B	1.171192	0.725131	0.040413	0.077*	
H30C	1.181538	0.914652	0.059459	0.077*	

### 4-[(2,3,4,5,6-Pentafluorophenoxy)carbonyl]phenyl 4-(decyloxy)benzoate Atomic displacement parameters (Å^2^)

**Table:**

	*U*^11^	*U*^22^	*U*^33^	*U*^12^	*U*^13^	*U*^23^
F1	0.073 (2)	0.047 (2)	0.0280 (18)	−0.0045 (18)	0.0051 (16)	−0.0068 (16)
F2	0.0429 (19)	0.057 (3)	0.051 (2)	−0.0179 (17)	0.0027 (15)	0.0054 (17)
F3	0.0466 (19)	0.062 (3)	0.047 (2)	−0.0015 (17)	0.0323 (16)	0.0047 (17)
F4	0.064 (2)	0.036 (2)	0.0286 (17)	−0.0027 (16)	0.0148 (15)	−0.0079 (15)
F5	0.0388 (17)	0.042 (2)	0.0380 (18)	−0.0093 (15)	0.0056 (14)	0.0129 (15)
O1	0.0321 (19)	0.034 (2)	0.028 (2)	0.0032 (17)	0.0069 (15)	0.0063 (16)
O2	0.041 (2)	0.045 (3)	0.029 (2)	0.0143 (18)	0.0178 (17)	0.0144 (18)
O3	0.0291 (18)	0.039 (2)	0.0236 (19)	−0.0033 (16)	0.0087 (15)	−0.0106 (16)
O4	0.036 (2)	0.045 (3)	0.031 (2)	−0.0128 (19)	0.0125 (16)	−0.0152 (18)
O5	0.0297 (19)	0.050 (3)	0.029 (2)	−0.0049 (17)	0.0105 (15)	−0.0048 (18)
C1	0.032 (3)	0.028 (3)	0.027 (3)	0.011 (2)	0.011 (2)	0.010 (2)
C2	0.046 (3)	0.036 (4)	0.019 (3)	0.003 (3)	0.010 (2)	0.001 (2)
C3	0.030 (3)	0.040 (4)	0.030 (3)	−0.008 (3)	0.004 (2)	0.006 (3)
C4	0.034 (3)	0.034 (4)	0.033 (3)	0.005 (3)	0.022 (2)	0.010 (3)
C5	0.039 (3)	0.023 (3)	0.026 (3)	−0.001 (2)	0.008 (2)	−0.003 (2)
C6	0.028 (3)	0.024 (3)	0.032 (3)	−0.002 (2)	0.004 (2)	0.007 (2)
C7	0.035 (3)	0.017 (3)	0.020 (3)	−0.001 (2)	0.009 (2)	0.001 (2)
C8	0.027 (3)	0.021 (3)	0.018 (3)	−0.003 (2)	0.006 (2)	−0.004 (2)
C9	0.028 (3)	0.020 (3)	0.018 (3)	0.004 (2)	0.002 (2)	0.001 (2)
C10	0.035 (3)	0.023 (3)	0.019 (3)	−0.002 (2)	0.009 (2)	0.003 (2)
C11	0.030 (3)	0.029 (3)	0.020 (3)	0.000 (2)	0.010 (2)	−0.006 (2)
C12	0.031 (3)	0.031 (4)	0.026 (3)	0.006 (2)	0.005 (2)	−0.004 (2)
C13	0.037 (3)	0.027 (3)	0.022 (3)	0.002 (2)	0.006 (2)	0.006 (2)
C14	0.033 (3)	0.016 (3)	0.035 (3)	0.002 (2)	0.009 (2)	0.003 (2)
C15	0.028 (3)	0.025 (3)	0.020 (3)	0.002 (2)	0.007 (2)	−0.005 (2)
C16	0.033 (3)	0.026 (3)	0.016 (3)	0.002 (2)	0.005 (2)	−0.002 (2)
C17	0.029 (3)	0.036 (4)	0.031 (3)	0.002 (2)	0.002 (2)	−0.003 (2)
C18	0.026 (3)	0.019 (3)	0.033 (3)	0.000 (2)	0.006 (2)	0.001 (2)
C19	0.031 (3)	0.041 (4)	0.020 (3)	−0.002 (2)	0.011 (2)	−0.004 (2)
C20	0.035 (3)	0.017 (3)	0.037 (3)	0.003 (2)	0.009 (2)	−0.003 (2)
C21	0.035 (3)	0.042 (4)	0.026 (3)	−0.004 (3)	0.011 (2)	−0.004 (2)
C22	0.031 (3)	0.044 (4)	0.029 (3)	0.002 (3)	0.006 (2)	−0.002 (3)
C23	0.037 (3)	0.051 (4)	0.022 (3)	−0.009 (3)	0.008 (2)	−0.002 (3)
C24	0.033 (3)	0.043 (4)	0.033 (3)	0.000 (3)	0.006 (2)	−0.001 (3)
C25	0.033 (3)	0.045 (4)	0.027 (3)	−0.002 (3)	0.010 (2)	−0.002 (3)
C26	0.039 (3)	0.049 (4)	0.029 (3)	−0.003 (3)	0.010 (2)	−0.006 (3)
C27	0.037 (3)	0.046 (4)	0.031 (3)	−0.004 (3)	0.008 (2)	−0.006 (3)
C28	0.037 (3)	0.054 (4)	0.032 (3)	−0.005 (3)	0.006 (2)	−0.005 (3)
C29	0.049 (3)	0.049 (4)	0.034 (3)	−0.005 (3)	0.014 (3)	−0.005 (3)
C30	0.053 (4)	0.067 (5)	0.040 (4)	−0.003 (3)	0.022 (3)	−0.009 (3)

### 4-[(2,3,4,5,6-Pentafluorophenoxy)carbonyl]phenyl 4-(decyloxy)benzoate Geometric parameters (Å, º)

**Table:**

F1—C2	1.351 (6)	C17—C18	1.403 (7)
F2—C3	1.340 (6)	C17—H17	0.9500
F3—C4	1.333 (5)	C18—C19	1.388 (7)
F4—C5	1.337 (6)	C19—C20	1.369 (7)
F5—C6	1.336 (6)	C19—H19	0.9500
O1—C7	1.192 (5)	C20—H20	0.9500
O2—O2	0.000 (11)	C21—C22	1.491 (7)
O2—C7	1.378 (6)	C21—H21A	0.9900
O2—C1	1.382 (6)	C21—H21B	0.9900
O3—O3	0.000 (7)	C22—C23	1.517 (7)
O3—C14	1.363 (6)	C22—H22A	0.9900
O3—C11	1.401 (5)	C22—H22B	0.9900
O4—C14	1.191 (6)	C23—C24	1.504 (7)
O5—C18	1.354 (6)	C23—H23A	0.9900
O5—C21	1.442 (6)	C23—H23B	0.9900
C1—C2	1.360 (7)	C24—C25	1.520 (7)
C1—C6	1.389 (7)	C24—H24A	0.9900
C2—C3	1.369 (7)	C24—H24B	0.9900
C3—C4	1.373 (7)	C25—C26	1.506 (7)
C4—C5	1.369 (7)	C25—H25A	0.9900
C5—C6	1.352 (7)	C25—H25B	0.9900
C7—C8	1.465 (6)	C26—C27	1.516 (7)
C8—C13	1.390 (7)	C26—H26A	0.9900
C8—C9	1.393 (6)	C26—H26B	0.9900
C9—C10	1.373 (6)	C27—C28	1.511 (7)
C9—H9	0.9500	C27—H27A	0.9900
C10—C11	1.365 (7)	C27—H27B	0.9900
C10—H10	0.9500	C28—C29	1.509 (7)
C11—C12	1.382 (7)	C28—H28A	0.9900
C12—C13	1.390 (7)	C28—H28B	0.9900
C12—H12	0.9500	C29—C30	1.520 (7)
C13—H13	0.9500	C29—H29A	0.9900
C14—C15	1.470 (7)	C29—H29B	0.9900
C15—C20	1.390 (7)	C30—H30A	0.9800
C15—C16	1.392 (7)	C30—H30B	0.9800
C16—C17	1.369 (7)	C30—H30C	0.9800
C16—H16	0.9500		
			
O2—O2—C7	0 (10)	O5—C18—C19	125.0 (4)
O2—O2—C1	0 (10)	O5—C18—C17	115.8 (4)
C7—O2—C1	117.8 (4)	C19—C18—C17	119.2 (4)
O3—O3—C14	0 (10)	C20—C19—C18	119.2 (5)
O3—O3—C11	0 (10)	C20—C19—H19	120.4
C14—O3—C11	117.9 (4)	C18—C19—H19	120.4
C18—O5—C21	117.7 (4)	C19—C20—C15	122.3 (5)
C2—C1—O2	122.6 (5)	C19—C20—H20	118.9
C2—C1—O2	122.6 (5)	C15—C20—H20	118.9
O2—C1—O2	0.0 (4)	O5—C21—C22	108.4 (4)
C2—C1—C6	117.9 (4)	O5—C21—H21A	110.0
O2—C1—C6	119.4 (5)	C22—C21—H21A	110.0
O2—C1—C6	119.4 (5)	O5—C21—H21B	110.0
F1—C2—C1	119.3 (5)	C22—C21—H21B	110.0
F1—C2—C3	119.1 (5)	H21A—C21—H21B	108.4
C1—C2—C3	121.5 (5)	C21—C22—C23	113.1 (4)
F2—C3—C2	120.1 (5)	C21—C22—H22A	109.0
F2—C3—C4	120.1 (5)	C23—C22—H22A	109.0
C2—C3—C4	119.7 (5)	C21—C22—H22B	109.0
F3—C4—C5	120.6 (5)	C23—C22—H22B	109.0
F3—C4—C3	120.1 (5)	H22A—C22—H22B	107.8
C5—C4—C3	119.3 (4)	C24—C23—C22	114.2 (4)
F4—C5—C6	120.5 (5)	C24—C23—H23A	108.7
F4—C5—C4	119.0 (4)	C22—C23—H23A	108.7
C6—C5—C4	120.4 (5)	C24—C23—H23B	108.7
F5—C6—C5	119.5 (5)	C22—C23—H23B	108.7
F5—C6—C1	119.5 (4)	H23A—C23—H23B	107.6
C5—C6—C1	121.0 (5)	C23—C24—C25	114.3 (4)
O1—C7—O2	121.2 (4)	C23—C24—H24A	108.7
O1—C7—O2	121.2 (4)	C25—C24—H24A	108.7
O2—C7—O2	0.0 (4)	C23—C24—H24B	108.7
O1—C7—C8	127.8 (4)	C25—C24—H24B	108.7
O2—C7—C8	111.0 (4)	H24A—C24—H24B	107.6
O2—C7—C8	111.0 (4)	C26—C25—C24	114.0 (4)
C13—C8—C9	119.9 (4)	C26—C25—H25A	108.7
C13—C8—C7	122.3 (4)	C24—C25—H25A	108.7
C9—C8—C7	117.7 (4)	C26—C25—H25B	108.7
C10—C9—C8	120.6 (4)	C24—C25—H25B	108.7
C10—C9—H9	119.7	H25A—C25—H25B	107.6
C8—C9—H9	119.7	C25—C26—C27	114.3 (4)
C11—C10—C9	118.8 (4)	C25—C26—H26A	108.7
C11—C10—H10	120.6	C27—C26—H26A	108.7
C9—C10—H10	120.6	C25—C26—H26B	108.7
C10—C11—C12	122.3 (4)	C27—C26—H26B	108.7
C10—C11—O3	120.2 (4)	H26A—C26—H26B	107.6
C12—C11—O3	117.3 (4)	C28—C27—C26	115.1 (4)
C10—C11—O3	120.2 (4)	C28—C27—H27A	108.5
C12—C11—O3	117.3 (4)	C26—C27—H27A	108.5
O3—C11—O3	0.0 (4)	C28—C27—H27B	108.5
C11—C12—C13	119.0 (5)	C26—C27—H27B	108.5
C11—C12—H12	120.5	H27A—C27—H27B	107.5
C13—C12—H12	120.5	C29—C28—C27	113.7 (4)
C12—C13—C8	119.3 (5)	C29—C28—H28A	108.8
C12—C13—H13	120.3	C27—C28—H28A	108.8
C8—C13—H13	120.3	C29—C28—H28B	108.8
O4—C14—O3	122.4 (4)	C27—C28—H28B	108.8
O4—C14—O3	122.4 (4)	H28A—C28—H28B	107.7
O3—C14—O3	0.0 (3)	C28—C29—C30	114.9 (5)
O4—C14—C15	125.7 (5)	C28—C29—H29A	108.6
O3—C14—C15	111.9 (4)	C30—C29—H29A	108.6
O3—C14—C15	111.9 (4)	C28—C29—H29B	108.6
C20—C15—C16	118.2 (4)	C30—C29—H29B	108.6
C20—C15—C14	118.9 (4)	H29A—C29—H29B	107.5
C16—C15—C14	122.9 (4)	C29—C30—H30A	109.5
C17—C16—C15	120.3 (5)	C29—C30—H30B	109.5
C17—C16—H16	119.8	H30A—C30—H30B	109.5
C15—C16—H16	119.8	C29—C30—H30C	109.5
C16—C17—C18	120.7 (5)	H30A—C30—H30C	109.5
C16—C17—H17	119.6	H30B—C30—H30C	109.5
C18—C17—H17	119.6		
			
O2—O2—C1—C2	0.0 (7)	C9—C10—C11—C12	1.8 (8)
C7—O2—C1—C2	78.7 (6)	C9—C10—C11—O3	−173.6 (4)
C7—O2—C1—O2	0 (100)	C9—C10—C11—O3	−173.6 (4)
O2—O2—C1—C6	0.0 (8)	O3—O3—C11—C10	0.0 (5)
C7—O2—C1—C6	−104.7 (5)	C14—O3—C11—C10	−70.8 (6)
O2—C1—C2—F1	−2.5 (8)	O3—O3—C11—C12	0.0 (6)
O2—C1—C2—F1	−2.5 (8)	C14—O3—C11—C12	113.5 (5)
C6—C1—C2—F1	−179.1 (5)	C14—O3—C11—O3	0 (100)
O2—C1—C2—C3	174.4 (5)	C10—C11—C12—C13	−2.2 (8)
O2—C1—C2—C3	174.4 (5)	O3—C11—C12—C13	173.4 (4)
C6—C1—C2—C3	−2.3 (8)	O3—C11—C12—C13	173.4 (4)
F1—C2—C3—F2	−2.0 (8)	C11—C12—C13—C8	1.4 (8)
C1—C2—C3—F2	−178.8 (5)	C9—C8—C13—C12	−0.3 (7)
F1—C2—C3—C4	177.4 (5)	C7—C8—C13—C12	−177.3 (5)
C1—C2—C3—C4	0.5 (8)	O3—O3—C14—O4	0.0 (11)
F2—C3—C4—F3	−0.9 (8)	C11—O3—C14—O4	−6.3 (7)
C2—C3—C4—F3	179.7 (5)	C11—O3—C14—O3	0 (100)
F2—C3—C4—C5	−179.9 (5)	O3—O3—C14—C15	0.0 (13)
C2—C3—C4—C5	0.8 (8)	C11—O3—C14—C15	171.9 (4)
F3—C4—C5—F4	−0.1 (8)	O4—C14—C15—C20	2.2 (8)
C3—C4—C5—F4	178.9 (5)	O3—C14—C15—C20	−176.0 (4)
F3—C4—C5—C6	−179.2 (5)	O3—C14—C15—C20	−176.0 (4)
C3—C4—C5—C6	−0.2 (8)	O4—C14—C15—C16	−178.4 (5)
F4—C5—C6—F5	−0.9 (8)	O3—C14—C15—C16	3.5 (7)
C4—C5—C6—F5	178.3 (5)	O3—C14—C15—C16	3.5 (7)
F4—C5—C6—C1	179.3 (5)	C20—C15—C16—C17	0.8 (8)
C4—C5—C6—C1	−1.6 (8)	C14—C15—C16—C17	−178.7 (5)
C2—C1—C6—F5	−177.0 (5)	C15—C16—C17—C18	0.8 (8)
O2—C1—C6—F5	6.2 (7)	C21—O5—C18—C19	11.8 (7)
O2—C1—C6—F5	6.2 (7)	C21—O5—C18—C17	−168.6 (4)
C2—C1—C6—C5	2.8 (8)	C16—C17—C18—O5	178.7 (5)
O2—C1—C6—C5	−174.0 (5)	C16—C17—C18—C19	−1.7 (8)
O2—C1—C6—C5	−174.0 (5)	O5—C18—C19—C20	−179.3 (5)
O2—O2—C7—O1	0.0 (14)	C17—C18—C19—C20	1.1 (8)
C1—O2—C7—O1	−3.6 (7)	C18—C19—C20—C15	0.5 (8)
C1—O2—C7—O2	0 (100)	C16—C15—C20—C19	−1.4 (8)
O2—O2—C7—C8	0.0 (16)	C14—C15—C20—C19	178.1 (5)
C1—O2—C7—C8	176.7 (4)	C18—O5—C21—C22	−178.6 (4)
O1—C7—C8—C13	−179.3 (5)	O5—C21—C22—C23	−176.3 (5)
O2—C7—C8—C13	0.4 (7)	C21—C22—C23—C24	−170.6 (5)
O2—C7—C8—C13	0.4 (7)	C22—C23—C24—C25	−177.8 (5)
O1—C7—C8—C9	3.6 (8)	C23—C24—C25—C26	−173.8 (5)
O2—C7—C8—C9	−176.7 (4)	C24—C25—C26—C27	−177.2 (5)
O2—C7—C8—C9	−176.7 (4)	C25—C26—C27—C28	−177.2 (5)
C13—C8—C9—C10	−0.1 (7)	C26—C27—C28—C29	−177.3 (5)
C7—C8—C9—C10	177.0 (4)	C27—C28—C29—C30	−175.2 (5)
C8—C9—C10—C11	−0.7 (7)		

### 4-[(2,3,4,5,6-Pentafluorophenoxy)carbonyl]phenyl 4-(decyloxy)benzoate Hydrogen-bond geometry (Å, º)

*Cg*3 and *Cg*2 are the centroids of the C15–C20 and C8–C13 rings, respectively.

**Table:**

*D*—H···*A*	*D*—H	H···*A*	*D*···*A*	*D*—H···*A*
C13—H13···O2	0.95	2.38	2.705 (6)	99
C16—H16···O3	0.95	2.42	2.737 (6)	99
C12—H12···F4^i^	0.95	2.48	3.427 (6)	174
C9—H9···O4^ii^	0.95	2.51	3.175 (7)	127
C3—F2···*Cg*3^iii^	1.34 (1)	3.24 (1)	3.630 (6)	96 (1)
C5—F4···*Cg*3^iv^	1.34 (1)	3.11 (1)	3.403 (6)	91 (1)
C6—F5···*Cg*2^v^	1.34 (1)	3.45 (1)	4.011 (6)	105 (1)

Symmetry codes: (i) −*x*+1, *y*−1/2, −*z*+5/2; (ii) −*x*+1, *y*+1/2, −*z*+3/2; (iii) −*x*+1, −*y*+1, −*z*+2; (iv) −*x*+1, −*y*+2, −*z*+2; (v) *x*, −*y*+3/2, *z*+1/2.
